# Temporal Sequence of Metabolic Syndrome Components: Abdominal Obesity as the Primary Driver of Progression in a Korean Prospective Cohort Study

**DOI:** 10.3390/medicina61081498

**Published:** 2025-08-21

**Authors:** Hoon Seok Kim, Jaeeun Yoo

**Affiliations:** 1 Department of Laboratory Medicine, Seoul St. Mary’s Hospital, College of Medicine, The Catholic University of Korea, Seoul 06591, Republic of Korea; 2 Department of Laboratory Medicine, Incheon St. Mary’s Hospital, College of Medicine, The Catholic University of Korea, Seoul 06591, Republic of Korea

**Keywords:** metabolic syndrome, transition patterns, central adiposity, prospective analysis

## Abstract

*Background and Objectives*: Metabolic syndrome (MetS) progresses gradually as individual components accumulate. However, there is limited understanding regarding whether the sequence of component appearance influences disease progression. This study sought to determine the most frequent initial MetS component and evaluate whether this component influences the subsequent risk of developing full MetS. *Materials and Methods*: We examined data from 6137 participants in the Korean Genome and Epidemiology Study (KoGES), free of MetS at baseline (2001–2002), followed until 2011–2012. Participants were stratified by the first emerging MetS component: abdominal obesity, elevated blood pressure, high fasting glucose, high triglycerides, or low HDL cholesterol. The primary endpoint was progression to full MetS, defined as the development of three or more components. We also assessed transition probabilities between components and sex-specific sequence differences. *Results*: Abdominal obesity was the most frequent initial metabolic abnormality (31.0%), followed by elevated blood pressure (26.3%), low HDL cholesterol (15.3%), high triglycerides (13.7%), and high fasting glucose (4.9%). Over a median 8.2-year follow-up, participants with initial abdominal obesity exhibited the greatest progression rate to full MetS (44.4%), significantly higher than those with elevated blood pressure (24.8%), high triglycerides (23.0%), high fasting glucose (21.6%), or low HDL cholesterol (9.3%) (all *p* < 0.001). After controlling for age, sex, smoking status, and baseline BMI, initial abdominal obesity was associated with a 4.77-fold increased risk (95% CI: 3.68–6.18) of developing full MetS compared to initial low HDL cholesterol. Distinct transition patterns were observed: high triglycerides frequently transitioned to low HDL cholesterol (78.1%), while abdominal obesity most often led to elevated blood pressure (52.1%). Marked sex-related differences were also found: abdominal obesity was more common initially among women (41.7% vs. 25.2%), whereas elevated blood pressure was predominant among men (37.6% vs. 21.2%). *Conclusions*: The initial MetS component strongly predicts progression to full syndrome, with abdominal obesity conferring the highest risk. Early identification and targeted interventions addressing abdominal obesity may effectively prevent MetS and its subsequent complications.

## 1. Introduction

Metabolic syndrome (MetS) is defined as a cluster of several cardiometabolic abnormalities that substantially elevate an individual’s risk of developing type 2 diabetes, cardiovascular disease, and mortality [1,2,3]. The diagnosis is established when an individual displays at least three out of five key features: abdominal obesity, elevated blood pressure, increased fasting glucose, elevated triglycerides, and reduced levels of high-density lipoprotein (HDL) cholesterol [4]. Although the clinical relevance of MetS is well-established, less consideration has been given to the progressive nature of its components, which typically emerge sequentially over time rather than appearing simultaneously [5].

Characterizing the temporal order in which MetS components manifest is critical for advancing our knowledge of disease progression and for designing more effective prevention strategies. While extensive research has addressed the prevalence of MetS and its associations with adverse clinical outcomes, relatively few investigations have examined the sequence in which each component develops [6,7,8]. This gap in the literature is particularly pronounced among Asian populations, who can display distinct MetS profiles compared to Western populations [9]. In particular, Korean populations have demonstrated unique characteristics, including lower BMI thresholds for risk, a higher prevalence of abdominal obesity and low HDL cholesterol, and evolving component patterns over time. For instance, Lim et al. [9] reported a significant increase in MetS prevalence in Korea from 1998 to 2007, driven primarily by rising rates of low HDL cholesterol and abdominal obesity, contrasting with trends in Western populations where blood pressure and glucose abnormalities are more prominent. These ethnic and temporal differences highlight the need for population-specific research to guide tailored prevention strategies and clarify variations in the sequence and progression of MetS components.

Recent studies further emphasize the complex interactions among genetic, environmental, and lifestyle determinants in influencing the pathogenesis of MetS [10]. Nonetheless, the order in which MetS components typically emerge remains uncertain, and it is not established whether the first-appearing component affects the rate or severity of syndrome progression. Additionally, the sequential development and transitions between individual components have not been thoroughly characterized, particularly in longitudinal cohort studies that monitor individuals over successive time points [11,12].

The Korean Genome and Epidemiology Study (KoGES) offers a unique opportunity to address these outstanding questions due to its comprehensive sample size, extended follow-up duration, and detailed longitudinal assessment of MetS components [13]. In this investigation, our objectives were: (i) to identify which MetS component most commonly presents initially, (ii) to assess whether the first emerging component impacts the risk of subsequent development of full MetS, (iii) to elucidate the patterns of transition among MetS components, and (iv) to determine whether sex differences exist in the developmental trajectories of these components.

By analyzing the temporal progression of MetS components, we seek to enhance understanding of syndrome evolution and to recognize early-stage high-risk markers.

## 2. Materials and Methods

### 2.1. Study Population

This study utilized data from the Korean Genome and Epidemiology Study (KoGES) [13], a large-scale, community-based prospective cohort established to investigate genetic and environmental factors contributing to chronic diseases among Korean adults. Initiated in 2001–2002, KoGES recruited participants aged 40 to 69 years from the Ansung and Ansan regions, and follow-up assessments were performed every two years. We selected individuals who attended at least three consecutive examination cycles (including baseline and two subsequent visits) to ensure comprehensive data on all five MetS components at each time point. Those diagnosed with MetS at baseline or lacking essential covariate information were excluded, resulting in a final analytical sample of 6137 participants. This study was approved by the Institutional Review Board of Incheon St. Mary’s Hospital (IRB number: OC25ZASI0053) and as this study used anonymized data that had already been completed, the requirement for obtaining additional informed consent was waived.

### 2.2. Definition of MetS Components

MetS components were determined using the revised National Cholesterol Education Program Adult Treatment Panel III criteria, incorporating Asian-specific waist circumference thresholds for abdominal obesity:

- Abdominal obesity (O): Waist circumference ≥ 90 cm for men or ≥85 cm for women;
- Elevated blood pressure (BP): Systolic blood pressure ≥ 130 mmHg or diastolic blood pressure ≥ 85 mmHg, or the use of antihypertensive medication;
- High fasting glucose (G): Fasting plasma glucose ≥ 100 mg/dL or the use of glucose-lowering medication;
- High triglycerides (T): Fasting triglycerides ≥ 150 mg/dL or the use of lipid-lowering medication;
- Low HDL cholesterol (H): HDL cholesterol < 40 mg/dL for men or <50 mg/dL for women.

MetS was characterized by the presence of three or more of these components. To assess the robustness of our findings, we also analyzed data using alternative criteria including the original NCEP ATP III thresholds and International Diabetes Federation (IDF) criteria, with results provided in the sensitivity analyses.

### 2.3. Anthropometric and Laboratory Measurements

Anthropometric measurements comprised height and weight (with participants in light clothing and barefoot) for BMI calculation (kg/m^2^), as well as waist circumference assessed at the midpoint between the lower rib and the iliac crest. Blood pressure was determined with a standard mercury sphygmomanometer in a seated position after a minimum of five minutes of rest. The mean of two separate readings was utilized for analysis. Fasting blood specimens (≥8 h) were used to quantify plasma glucose, total cholesterol, triglycerides, and HDL cholesterol using an automated analyzer (ADVIA 1650; Siemens, Tarrytown, NY, USA).

### 2.4. Assessment of Temporal Sequence and Transitions

For each participant, we identified the first MetS component to present during follow-up and grouped participants based on this initial manifestation. Participants who simultaneously developed more than one MetS component at a single examination (*n* = 547, 8.9%) were excluded from primary analyses to ensure a precise assessment of temporal order. This exclusion was necessary to clearly delineate a single initial MetS component and its direct impact on subsequent progression, which could be confounded if multiple components appeared simultaneously. To address potential bias from this exclusion, we conducted sensitivity analyses including this subgroup, which confirmed that our primary conclusions remain robust. To investigate how MetS components emerged over time, we monitored the consecutive development of additional components among individuals with a single initial component. Transition probabilities were estimated by dividing the number of specific transitions between components by the total number of transitions from the same starting component.

### 2.5. Assessment of Outcomes

The primary outcome was defined as progression to full MetS, characterized by the development of at least three MetS components during follow-up. Among the 5590 individuals with a single initial MetS component, 4365 were included in progression analyses after excluding those who were lost to follow-up or censored before the endpoint could be ascertained. As an exploratory secondary outcome, we measured the incidence of diabetes, defined by any of the following: fasting plasma glucose ≥ 126 mg/dL, 2 h plasma glucose ≥ 200 mg/dL during a 75 g oral glucose tolerance test, HbA1c ≥ 6.5%, or the initiation of glucose-lowering therapy.

### 2.6. Statistical Analysis

We compared baseline characteristics between groups using analysis of variance for continuous variables and chi-square tests for categorical variables. MetS progression rates were computed by initial component group and compared using chi-square tests, applying Bonferroni correction for multiple testing. Transition probabilities among MetS components were expressed as percentages and included 95% CIs. Chi-square tests determined whether the observed transitions significantly deviated from random chance. Prior to descriptive analysis, the distribution of continuous variables was evaluated using the Shapiro–Wilk test and visual inspection of histograms. As most variables were approximately normally distributed, results are presented as mean ± standard deviation (SD).

Associations between the initial metabolic component and the likelihood of progression to full MetS were evaluated using multivariable logistic regression models, adjusting for age, sex, smoking status, and baseline BMI; results are reported as odds ratios (ORs) with 95% confidence intervals (CIs). To assess risk of incident diabetes, Cox proportional hazards models were constructed with adjustment for age, sex, smoking, family history of diabetes, and baseline BMI; hazard ratios (HRs) with 95% CIs are presented.

We included baseline BMI as a covariate in our models to control for potential confounding by general obesity. Given concerns about possible collinearity or over-adjustment when assessing the impact of abdominal obesity, we conducted additional sensitivity analyses without adjusting for baseline BMI to evaluate the robustness of our findings. Multicollinearity was assessed using correlation coefficients and variance inflation factors (VIF), with VIF values > 5.0 considered indicative of problematic multicollinearity.

Effect sizes were calculated to quantify the magnitude of observed associations and differences beyond statistical significance. For categorical analyses, Cramer’s V was used for associations involving variables with more than two categories, and phi coefficients were calculated for binary associations. For continuous variables, Cohen’s d was computed to assess standardized mean differences between groups. Effect sizes were interpreted according to conventional guidelines: small (Cramer’s V: 0.1–0.29; Cohen’s d: 0.2–0.49), medium (Cramer’s V: 0.3–0.49; Cohen’s d: 0.5–0.79), and large (Cramer’s V: ≥0.5; Cohen’s d: ≥0.8) effects.

All analyses were performed using R version 4.0.3 (R Foundation, Vienna, Austria), and statistical significance was set at two-sided *p*-values < 0.05.

## 3. Results

### 3.1. Baseline Characteristics

Among the 6137 participants analyzed, abdominal obesity was most frequently identified as the first MetS component, present in 31.0% (*n* = 1902) of individuals. This was followed by elevated blood pressure (26.3%, *n* = 1611), high triglycerides (13.7%, *n* = 839), high fasting glucose (4.9%, *n* = 302), and low HDL cholesterol (15.3%, *n* = 936). A minority of participants (8.9%, *n* = 547) developed multiple components concurrently and were therefore excluded from the main analyses.

Table 1 presents the baseline characteristics of study participants categorized by their initial MetS component. Statistically significant differences were found between groups for all assessed characteristics (all *p* < 0.001). Participants with abdominal obesity as the initial component were predominantly female (63.2%) and had a higher mean BMI (25.9 ± 2.8 kg/m^2^). In contrast, those whose initial component was elevated blood pressure tended to be older (mean age: 54.1 ± 8.8 years) and predominantly male (62.2%). Individuals who began with low HDL cholesterol as their initial component were generally younger (mean age: 49.5 ± 8.1 years) and exhibited a lower mean BMI (22.7 ± 2.4 kg/m^2^).

### 3.2. Progression to MetS by Initial Component

Over a median follow-up of 8.2 years (interquartile range: 7.6–8.8 years), MetS was diagnosed in 1105 participants (18.0%). Rates of progression to MetS varied significantly according to the initial MetS component (χ^2^ = 320.2, df = 4, *p* < 0.0001). The effect size for this association was large (Cramer’s V = 0.365), indicating substantial differences in progression rates between initial component groups.

**Figure 1:**
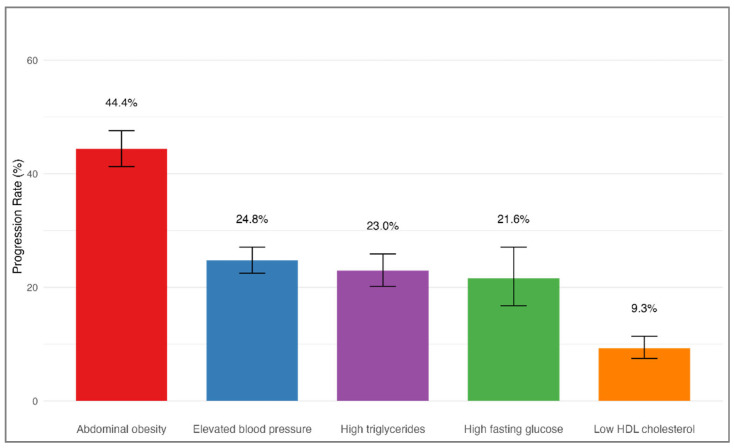
Progression to MeS by initial component. Progression rates to MetS during follow-up stratified by the initially identified MetS component (*n* = 4365). Participants who initially presented with abdominal obesity exhibited the highest progression rate (44.4%), followed by elevated blood pressure, high triglycerides, high fasting glucose, and low HDL cholesterol. Error bars represent 95% confidence intervals. Group differences were assessed using the chi-square test (*p* < 0.001).

Table 2 and Figure 1 demonstrate that those with abdominal obesity as the first component experienced the highest MetS progression rate at 44.4% (95% CI: 41.3–47.6%). Participants whose initial findings were elevated blood pressure progressed at 24.8% (95% CI: 22.5–27.1%), followed by high triglycerides at 23.0% (95% CI: 20.2–25.9%), high fasting glucose at 21.6% (95% CI: 16.8–27.1%), and those with low HDL cholesterol at 9.3% (95% CI: 7.5–11.4%).

Following adjustment for age, sex, smoking status, and baseline BMI, individuals with abdominal obesity as their initial component exhibited 4.77-fold higher odds (95% CI: 3.68–6.18) of developing MetS relative to those initially presenting with low HDL cholesterol (reference group). Likewise, those with elevated blood pressure, high triglycerides, or high fasting glucose as the initial component showed increased odds of 2.67-fold (95% CI: 2.05–3.47), 2.48-fold (95% CI: 1.86–3.30), and 2.32-fold (95% CI: 1.58–3.41), respectively (all *p* < 0.001).

### 3.3. Sex Differences in Initial Component Distribution

**Figure 2:**
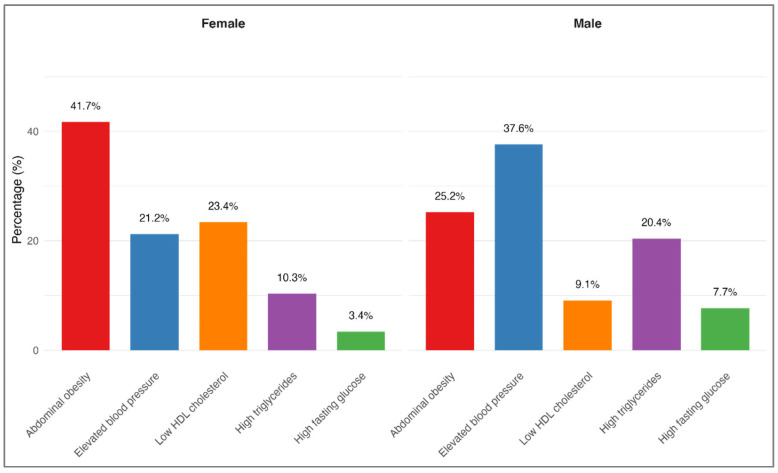
Sex differences in distribution of initial MetS components. Distribution of the first identified MetS components by sex in the study population (*n* = 4365). Among women, abdominal obesity (41.7%) and low HDL cholesterol (23.4%) were the most frequent initial abnormalities. Among men, elevated blood pressure (37.6%) and abdominal obesity (25.2%) were most common. Group differences were assessed using chi-square tests (χ^2^ = 551.7, df = 4, *p* < 0.0001). Error bars represent 95% confidence intervals.

Men and women differed significantly in the distribution of the first MetS component to appear (χ^2^ = 551.7, df = 4, *p* < 0.0001). The overall effect size for sex differences was large (Cramer’s V = 0.314), with particularly strong sex-based patterns observed for abdominal obesity (Cramer’s V = 0.357) and low HDL cholesterol (Cramer’s V = 0.260). As illustrated in Figure 2, abdominal obesity was the most common initial component among female participants (41.7%), followed by low HDL cholesterol (23.4%) and elevated blood pressure (21.2%). In contrast, among male participants, elevated blood pressure was most frequently the first component (37.6%), with abdominal obesity (25.2%) and high triglycerides (20.4%) presenting next in frequency. After adjustment for age, BMI, and smoking status, these sex-specific distributions remained statistically significant (all *p* < 0.001), suggesting distinct development patterns of MetS between men and women.

### 3.4. Transition Patterns Between Components

**Figure 3:**
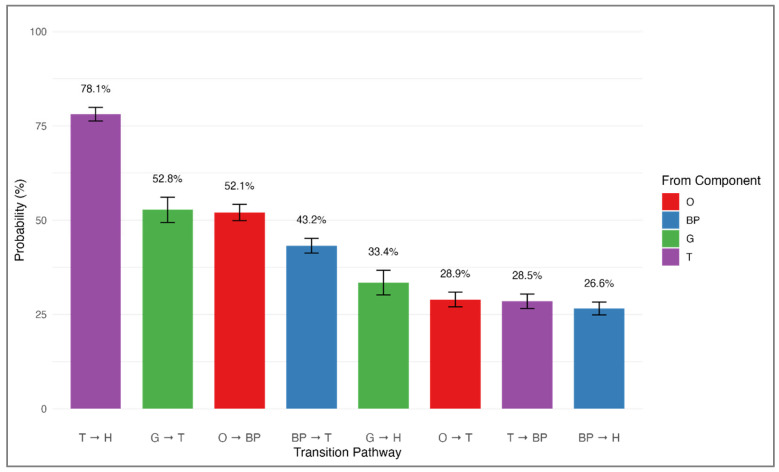
Most common transition probabilities between MetS components. Bar plot showing the top eight most frequent sequential transitions between individual MetS components during follow-up among participants who initially had a single component (*n* = 5590). Transition probabilities were calculated based on observed transitions relative to all possible transitions from each initial component. The most common transition was from T to H (78.1%), followed by transitions such as G to T, and O to BP. Error bars represent 95% confidence intervals. Statistical significance for each transition was assessed using chi-square tests (all *p* < 0.001). Abbreviations: O, abdominal obesity; BP, elevated blood pressure; T, high triglycerides; G, high fasting glucose; H, low HDL cholesterol.

**Figure 4:**
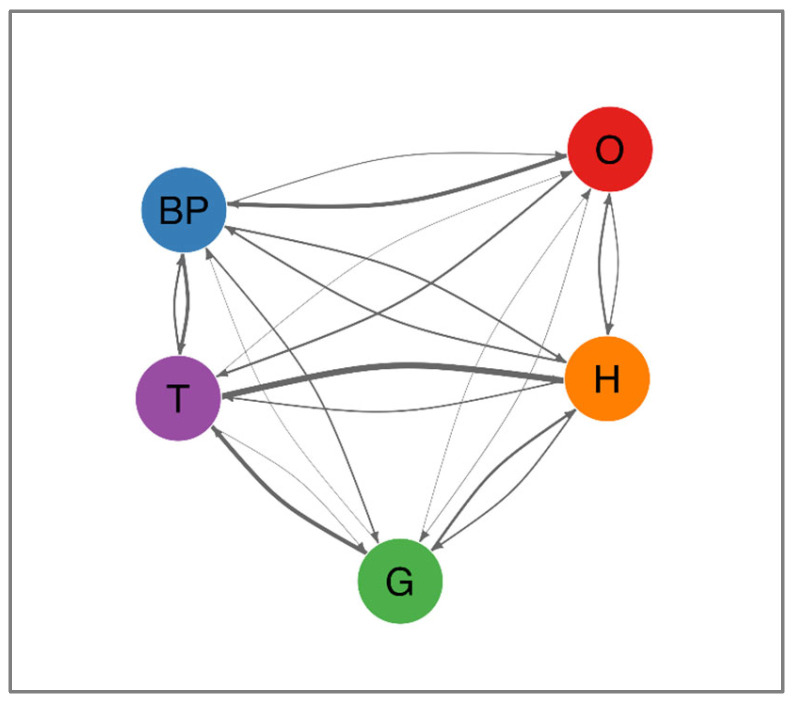
Network diagram of transitions between MetS components. This network diagram depicts the directional transitions between metabolic syndrome (MetS) components among participants with a single initial component (*n* = 5590). Each node represents a MetS component, and arrows indicate the direction of progression to another component. The thickness of each arrow reflects the observed transition probability between the corresponding pair of components, derived from longitudinal follow-up data. Thicker arrows represent more frequent transitions. Notably, transitions from T to H and from O to BP were among the most common. Abbreviations: O, abdominal obesity; BP, elevated blood pressure; T, high triglycerides; G, high fasting glucose; H, low HDL cholesterol.

When analyzing the patterns by which MetS components developed over time, we determined that transitions between components were not random but instead followed distinct, statistically significant trajectories (all *p* < 0.0001). The overall effect size for non-random transition patterns was large (Cramer’s V = 0.428), confirming that MetS components follow structured rather than random developmental pathways. Table 3 summarizes the most frequent transitions, which are further depicted in Figure 3. Notably, the transition from high triglycerides to low HDL cholesterol was the most prevalent (78.1%, 95% CI: 76.3–79.9%). Additional common progressions included high fasting glucose advancing to high triglycerides (52.8%, 95% CI: 49.4–56.1%), abdominal obesity progressing to elevated blood pressure (52.1%, 95% CI: 49.9–54.2%), and elevated blood pressure shifting to high triglycerides (43.2%, 95% CI: 41.3–45.2%). A comprehensive depiction of these and other less frequent pathways is provided in Figure 4, which presents a network diagram visualizing all observed transitions between MetS components. The directional arrows illustrate the flow of progression, with their thickness reflecting transition probabilities. Notably, components such as high triglycerides and abdominal obesity exhibited multiple outbound transitions, indicating their frequent involvement as initiating or intermediary abnormalities. Collectively, these data suggest that metabolic disturbances tend to occur in consistent sequences rather than randomly.

### 3.5. Component Co-Occurrence at Baseline

**Figure 5:**
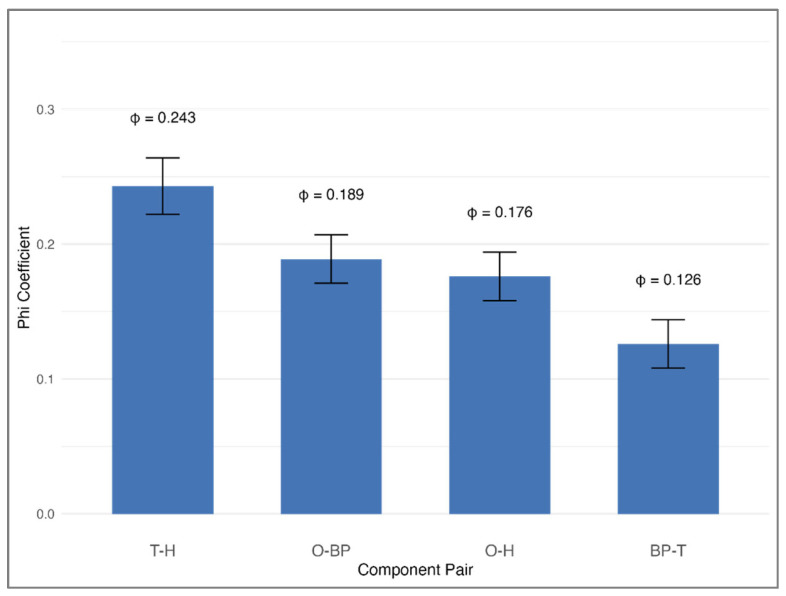
Pairwise associations between MetS components at baseline (phi coefficients). Bar plot showing phi coefficients for the most significant pairwise associations between MetS components at baseline among all study participants (*n* = 6137). The strongest correlation was between T and H (φ = 0.243), followed by associations between O and BP, and between O and H. Error bars denote 95% confidence intervals. Abbreviations: O, abdominal obesity; BP, elevated blood pressure; T, high triglycerides; G, high fasting glucose; H, low HDL cholesterol.

In evaluating the frequency with which specific pairs of MetS components co-occurred at baseline, we identified clear and statistically significant clustering that deviated from random distribution (all *p* < 0.0001; Figure 5). The strongest relationship was observed between high triglycerides and low HDL cholesterol (phi coefficient = 0.243, representing a medium effect size). Significant pairings also included abdominal obesity with elevated blood pressure (phi = 0.189), abdominal obesity with low HDL cholesterol (phi = 0.176), and elevated blood pressure with high triglycerides (phi = 0.126). These results underscore that certain metabolic abnormalities tend to co-occur rather than arise independently, supporting the hypothesis that overlapping biological processes may underlie their simultaneous presentation.

### 3.6. Exploratory Analysis: Diabetes Incidence by Initial Component

**Figure 6:**
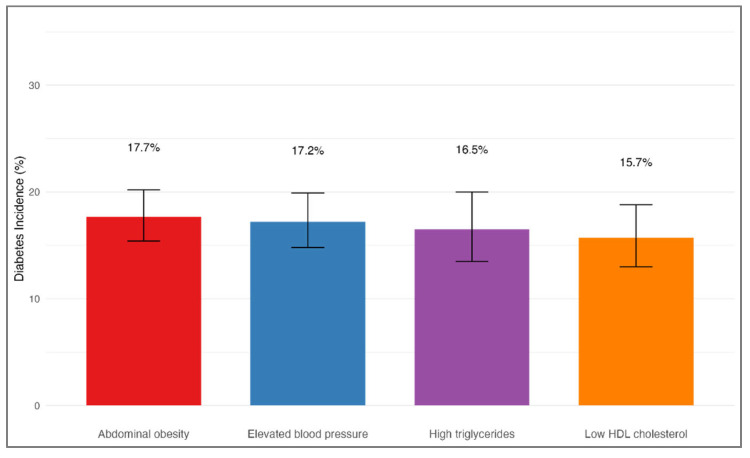
Incidence of diabetes by initial MetS component. Bar plot illustrating the incidence of diabetes during the follow-up period, stratified by the first emerging MetS component among participants (*n* = 4365). The incidence rates were as follows: 17.7% for those with initial abdominal obesity, 17.2% for elevated blood pressure, 16.5% for high triglycerides, and 15.7% for low HDL cholesterol. Differences between the initial components were not statistically significant (*p* = 0.751). Error bars denote 95% confidence intervals.

Among participants who did not have diabetes at baseline, 523 individuals (16.9%) were diagnosed with diabetes during the follow-up period. The incidence of diabetes did not significantly differ based on the initial MetS component (χ^2^ = 1.2, df = 3, *p* = 0.751), as shown in Table 4 and Figure 6. Specifically, the diabetes incidence was 17.7% (95% CI: 15.4–20.2%) among individuals whose initial component was abdominal obesity, 17.2% (95% CI: 14.8–19.9%) for those with elevated blood pressure, 16.5% (95% CI: 13.5–20.0%) among those with high triglycerides, and 15.7% (95% CI: 13.0–18.8%) for participants with low HDL cholesterol at baseline. After adjusting for age, sex, smoking status, family history of diabetes, and baseline BMI, the type of initial MetS component remained unassociated with the likelihood of developing diabetes. Using the group with elevated blood pressure as the reference, the adjusted HRs were 1.03 (95% CI: 0.82–1.29) for abdominal obesity, 0.96 (95% CI: 0.74–1.25) for high triglycerides, and 0.92 (95% CI: 0.71–1.18) for low HDL cholesterol (all *p* > 0.05).

### 3.7. Sensitivity Analyses

Several sensitivity analyses were conducted to assess the robustness of our primary findings. First, we repeated the main analyses by including participants with simultaneous onset of multiple MetS components (*n* = 547), designating them as a distinct category. The findings remained largely unchanged, with progression patterns to MetS consistent across groups. We also assessed alternative MetS definitions, incorporating both the IDF and original NCEP ATP III criteria without Asian-specific waist circumference cutoffs. While there were minor adjustments to progression rates, the relative order of progression by initial component was aligned with the main results. In another analysis, we restricted the population to participants with at least 4 years of follow-up to allow sufficient time for MetS development, and observed results that mirrored our initial findings. Finally, we examined whether associations between initial MetS component and progression differed by age, sex, or baseline BMI. Although minor subgroup differences in association strength were noted, the overall progression trends were stable and support the generalizability of our main findings across demographic and clinical strata. Sensitivity analyses excluding BMI from logistic regression models showed virtually identical results (abdominal obesity OR: 13.692 vs. 13.687 with BMI adjustment), with minimal correlation between waist circumference and BMI (r = −0.002), confirming the stability of our findings and absence of problematic multicollinearity.

Analysis using alternative MetS criteria demonstrated the robustness of our findings. Compared to our primary analysis using NCEP ATP III with Asian-specific waist cutoffs (38.6% baseline prevalence, 8.9% progression rate), the original NCEP ATP III criteria yielded lower prevalence (30.7%) and progression rates (4.2%) due to higher waist circumference thresholds, while IDF criteria showed similar patterns (27.4% prevalence, 9.9% progression rate). Despite these quantitative differences, abdominal obesity consistently emerged as the predominant initial component across all criteria, confirming the robustness of our conclusions regarding MetS developmental pathways.

## 4. Discussion

In this large-scale, prospective cohort study of Korean adults, we identified distinct and meaningful sequences in the emergence of MetS components, which substantially influenced the probability of progressing to full MetS. Abdominal obesity was the most frequent initial component, followed sequentially by elevated blood pressure, low HDL cholesterol, high triglycerides, and lastly high fasting glucose. Importantly, the nature of the first metabolic abnormality markedly affected progression risk; individuals with abdominal obesity as their initial abnormality exhibited nearly five times higher odds of developing full MetS than those whose first abnormality was low HDL cholesterol. Moreover, we observed significant sex-based differences in the initial component presentation and elucidated progression pathways, supporting that the development of MetS follows a structured rather than random course. These results not only reaffirm the central role of abdominal obesity but also extend current understanding by demonstrating that the sequence in which MetS components emerge—particularly when abdominal obesity appears first—has a substantial impact on the progression trajectory. By focusing on temporal patterns rather than isolated components, our findings offer novel, clinically actionable insights into early risk stratification, especially for Asian populations where MetS profiles may differ from those observed in Western settings.

The finding that abdominal obesity most often appeared first aligns with its recognized central role in MetS pathogenesis. Several prior longitudinal investigations have reported similar patterns [14,15,16]. For instance, Haring et al. [14] used SHIP cohort data from Germany and demonstrated that central obesity was the first component in approximately half of those progressing to MetS. Likewise, Hwang et al. [15], in a Korean community-based study, observed abdominal obesity as the most frequent initial abnormality. Our findings build on these prior reports by confirming that while abdominal obesity is usually the first component to manifest, it also significantly raises the likelihood of progressing to full MetS, as indicated by a notably high progression rate of 44.4% in comparison to other initial components.

The high predictive value of abdominal obesity as an initial component likely underscores its key involvement in the underlying mechanisms of MetS. Impaired visceral adipose tissue leads to insulin resistance, persistent low-grade inflammation, and dyslipidemia through pathways including increased free fatty acid delivery to the liver and alterations in adipokine secretion [17]. Sattar et al. [18] proposed that excessive central fat deposition may act as a “common soil,” fostering the development of other metabolic abnormalities. Our observations, notably the frequent transition from abdominal obesity to elevated blood pressure (52.1%) and high triglycerides (28.9%), provide additional evidence for the concept of metabolic cascades initiated by central adiposity. Notably, contemporary research has introduced metabolic indices such as the visceral adiposity index (VAI) and dysfunctional adiposity index (DAI) to better capture the degree of visceral fat accumulation and adipose tissue impairment, thereby emphasizing the intricate association between central adiposity and insulin resistance [19]. However, this study did not incorporate direct assessments of visceral adiposity, such as imaging modalities or surrogate indices like the visceral adiposity index (VAI). As a result, while abdominal obesity was defined using waist circumference, which reflects both subcutaneous and visceral fat, the precise mechanistic contribution of visceral fat accumulation could not be determined. Future studies using direct or surrogate measures of visceral adiposity may help clarify the pathophysiological role of central fat in MetS progression.

The distinct differences observed between men and women in their initial MetS components may be attributed to fundamental variations in body fat distribution and cardiometabolic risk profiles associated with sex. In our cohort, women predominantly first exhibited abdominal obesity (41.7%) or low HDL cholesterol (23.4%), while men more commonly presented with elevated blood pressure (37.6%) or high triglycerides (20.4%) as their initial component. These results are consistent with prior findings from the DESIR cohort [20], which demonstrated that menopause-associated alterations in fat distribution affect MetS component clustering among women. Such sex-specific differences may partially be due to hormonal influences, especially the regulatory effects of estrogen on both fat distribution and lipid metabolism [21]. Comparable patterns in the clustering and composition of MetS components by sex have been documented in another Korean cohort, underlining the significance of these patterns in determining the effectiveness of lifestyle modification interventions, and highlighting the potential benefit of component-specific strategies for prevention [22].

Our analysis of transition probabilities revealed that certain metabolic abnormalities frequently emerge in tandem. Notably, we identified the strongest association between high triglycerides and low HDL cholesterol, with a transition probability of 78.1%. This observation corroborates the current understanding of dyslipidemia, where elevated triglycerides promote accelerated HDL particle catabolism and alter HDL composition [23]. Furthermore, the frequent progression from abdominal obesity to elevated blood pressure (52.1%) observed in this study reflects well-established mechanistic links between increased adiposity and the development of hypertension. This association may be mediated by several physiological pathways, including augmented sympathetic nervous system activity, stimulation of the renin–angiotensin–aldosterone system, and impaired endothelial function [24].

Interestingly, while the initial type of MetS component was a strong predictor for progression to full MetS, it did not significantly influence the risk of developing diabetes. Although this was not the primary focus of our study, we performed an exploratory analysis to evaluate whether the initial MetS component could predict diabetes risk. Diabetes incidence remained relatively consistent across different groups, ranging from 15.7% to 17.7% (*p* = 0.751). This finding indicates that, for diabetes prevention, the detection of any metabolic abnormality should lead to prompt intervention, regardless of the sequence in which abnormalities present. Although previous studies have suggested that diabetes risk may vary depending on the type of initial metabolic abnormality [25], our results support more recent literature indicating that the total number or burden of metabolic abnormalities may be more critical than their specific order for assessing diabetes risk [26]. Our finding contrasts with some previous reports, and possible explanations for this discrepancy include differences in study populations, definitions used for incident diabetes, follow-up durations, and sample sizes. Specifically, our cohort was composed of middle-aged Korean adults, and the relatively uniform diabetes incidence across different initial MetS components may reflect that the total metabolic burden, rather than the sequence of emergence, predominantly drives diabetes risk. Future studies with larger, diverse populations and longer follow-up periods are warranted to further clarify this association.

Beyond examining the sequence and transitions of traditional MetS components, recent studies have investigated the addition of novel metabolic markers to refine MetS diagnostic criteria and improve risk prediction accuracy. Zhang et al. [27] proposed adding hyperuricemia as a sixth criterion to the standard MetS definition, demonstrating enhanced predictive value for long-term cardiovascular outcomes and mortality. Recognizing more at-risk individuals through this expanded framework reflects the evolving perspective on MetS, emphasizing the necessity for ongoing diagnostic updates to enable more precise and effective preventive interventions.

Our results have several key implications for both clinical practice and public health initiatives. Firstly, prevention programs should prioritize reducing abdominal obesity, especially in populations where it is commonly observed as the first abnormality. Secondly, the strong prognostic significance of the initial metabolic abnormality means that individuals initially identified with abdominal obesity require particularly close monitoring and earlier, intensive intervention to minimize the risk of subsequent metabolic disorders. In practical terms, routine screening for abdominal obesity using waist circumference, a simple and inexpensive method, could facilitate early detection. This would allow for timely lifestyle interventions, such as improving diet and increasing physical activity, particularly in resource-limited settings. Thirdly, the significant sex differences observed in the progression of metabolic abnormalities suggest that designing sex-specific preventive strategies may be advantageous.

### Limitations

This study has several limitations that warrant consideration. First, our analysis was conducted exclusively in a middle-aged Korean population, which may limit the generalizability of the findings to other ethnic groups or different age populations due to potential variations in genetic factors, lifestyle, dietary habits, and socioeconomic conditions. Second, the exclusion of participants with simultaneous onset of multiple components, while necessary for temporal clarity, may introduce selection bias by omitting individuals with potentially distinct metabolic trajectories, though sensitivity analyses confirmed the robustness of our main findings. Third, although the study employed a longitudinal design, the two-year interval between follow-up examinations might have obscured the precise temporal sequence or the simultaneous onset of MetS components, potentially introducing misclassification bias. Fourth, the definitions of MetS and its components rely on somewhat arbitrary threshold values; nonetheless, sensitivity analyses employing alternative criteria yielded consistent results. Lastly, while our analytical approach captured progression patterns and transition probabilities, more advanced modeling techniques—such as time-to-event analysis or transition matrix modeling—may offer deeper insights into the dynamic interrelations between MetS components. Future research incorporating more frequent assessments and involving more diverse populations is recommended to accurately determine the sequence of MetS component emergence and enhance the external validity of the findings.

## 5. Conclusions

In conclusion, our study demonstrates that MetS arises via distinct sequences of component accumulation, with the initial component exerting a substantial effect on progression risk. Notably, abdominal obesity emerged as the most influential factor driving MetS development, supporting the concept of metabolic disturbances triggered by excess adiposity. These results underscore the importance of early identification of abdominal obesity and suggest that focused interventions targeting central adiposity could substantially reduce the risk of full MetS and related adverse health outcomes.

## Figures and Tables

**Table 1:** Baseline characteristics of study participants by initial MetS component (*n* = 5590).

Characteristics	Abdominal Obesity (O)	Elevated Blood Pressure (BP)	High Triglycerides (T)	High Fasting Glucose (G)	Low HDL Cholesterol (H)	*p*-Value
*n* (%)	1902 (31.0)	1611 (26.3)	839 (13.7)	302 (4.9)	936 (15.3)	-
Age, years	52.4 ± 8.7	54.1 ± 8.8	51.2 ± 8.3	53.8 ± 9.1	49.5 ± 8.1	<0.001
Sex, *n* (%)						<0.001
Female	1203 (63.2)	609 (37.8)	295 (35.2)	98 (32.5)	672 (71.8)	
Male	699 (36.8)	1002 (62.2)	544 (64.8)	204 (67.5)	264 (28.2)	
BMI, kg/m^2^	25.9 ± 2.8	23.8 ± 2.6	24.2 ± 2.7	24.1 ± 2.5	22.7 ± 2.4	<0.001
Waist circumference, cm	88.7 ± 6.9	80.2 ± 7.3	82.4 ± 7.1	82.1 ± 6.8	77.3 ± 6.4	<0.001
Systolic BP, mmHg	119.4 ± 17.2	138.7 ± 14.6	121.8 ± 16.3	120.3 ± 16.8	116.2 ± 15.1	<0.001
Diastolic BP, mmHg	79.2 ± 10.7	89.6 ± 9.8	79.8 ± 10.3	78.9 ± 10.4	77.1 ± 9.8	<0.001
Fasting glucose, mg/dL	87.3 ± 8.6	88.6 ± 8.9	89.1 ± 8.7	112.7 ± 11.7	86.4 ± 8.2	<0.001
Triglycerides, mg/dL	119.4 ± 58.2	126.8 ± 56.7	192.3 ± 41.8	132.6 ± 54.9	116.2 ± 55.3	<0.001
HDL cholesterol, mg/dL	51.7 ± 11.2	50.3 ± 10.8	48.2 ± 10.7	49.8 ± 10.4	38.5 ± 6.3	<0.001
Current smoker, *n* (%)	389 (20.5)	452 (28.1)	286 (34.1)	94 (31.1)	166 (17.7)	<0.001

Baseline characteristics of participants grouped according to the first emerging metabolic syndrome (MetS) component during follow-up. Continuous variables are presented as mean ± standard deviation (SD), and categorical variables as number (%). Comparisons across groups were performed using one-way analysis of variance (ANOVA) for continuous variables and chi-square tests for categorical variables. Effect size for overall group differences: Cramer’s V = 0.314 (large effect). Abbreviations: O, abdominal obesity; BP, elevated blood pressure; T, high triglycerides; G, high fasting glucose; H, low HDL cholesterol; HDL, high-density lipoprotein; BMI, body mass index.

**Table 2:** Progression to MetS according to initial component (*n* = 4365).

Initial Component	Total (*n*)	Progressed to MetS (*n*)	Progression Rate (%)	95% CI (%)	Adjusted OR * (95% CI)	*p*-Value
Abdominal obesity (O)	977	434	44.4	41.3–47.6	4.77 (3.68–6.18)	<0.001
Elevated blood pressure (BP)	1345	333	24.8	22.5–27.1	2.67 (2.05–3.47)	<0.001
High triglycerides (T)	839	193	23.0	20.2–25.9	2.48 (1.86–3.30)	<0.001
High fasting glucose (G)	268	58	21.6	16.8–27.1	2.32 (1.58–3.41)	<0.001
Low HDL cholesterol (H)	936	87	9.3	7.5–11.4	1.00 (reference)	-

Progression rates to metabolic syndrome (MetS) among participants stratified by the first emerging MetS component during follow-up. The total number of participants, number who progressed to MetS, corresponding rates with 95% confidence intervals (CIs), and adjusted odds ratios (ORs) with 95% CIs are shown. * Odds ratios (ORs) are adjusted for age, sex, smoking status, and baseline body mass index (BMI). Multivariable logistic regression models were adjusted for age, sex, smoking status, and baseline body mass index (BMI).

**Table 3:** Most common transition probabilities between MetS components (*n* = 5590).

Transition Trajectory	Transition Probability (%)	95% CI (%)	Number of Observed Transitions (*n*)	*p*-Value *	Effect Size (Cramer’s V)
T_→_H	78.1	76.3–79.9	1684/2155	<0.001	0.624 (Large)
G_→_T	52.8	49.4–56.1	449/851	<0.001	0.458 (Large)
O_→_BP	52.1	49.9–54.2	1112/2136	<0.001	0.412 (Large)
BP_→_T	43.2	41.3–45.2	1070/2475	<0.001	0.385 (Large)
G_→_H	33.4	30.2–36.7	284/851	<0.001	0.298 (Medium)
O_→_T	28.9	27.0–30.9	617/2136	<0.001	0.276 (Medium)
T_→_BP	28.5	26.6–30.4	614/2155	<0.001	0.265 (Medium)
BP_→_H	26.6	24.9–28.3	658/2475	<0.001	0.248 (Medium)

The eight most frequently observed sequential transitions between individual metabolic syndrome (MetS) components among participants who initially presented with a single component. Transition probabilities were calculated as the proportion of specific transitions relative to all transitions originating from the same initial component. 95% confidence intervals (CIs) and *p*-values from chi-square tests are provided. * *p*-values were obtained from chi-square tests comparing observed transition frequencies with expected frequencies under the assumption of random distribution. Effect sizes represent the strength of each transition pattern deviation from random occurrence. Abbreviations: O, abdominal obesity; BP, elevated blood pressure; T, high triglycerides; G, high fasting glucose; H, low HDL cholesterol.

**Table 4:** Incidence of diabetes by initial MetS component (*n* = 4365).

Initial State	Total Population (*n*)	Number of Diabetes Cases (*n*)	Incidence Rate (%)	95% CI (%)	Adjusted HR (95% CI)	*p*-Value
Abdominal obesity (O)	987	175	17.7	15.4–20.2	1.03 (0.82–1.29)	0.82
Elevated blood pressure (BP)	912	157	17.2	14.8–19.9	1.00 (reference)	-
High triglycerides (T)	538	89	16.5	13.5–20.0	0.96 (0.74–1.25)	0.76
Low HDL cholesterol (H)	648	102	15.7	13.0–18.8	0.92 (0.71–1.18)	0.5

Diabetes incidence during follow-up stratified by the first-appearing MetS component at baseline. Incidence rates are shown with 95% confidence intervals (CIs). Adjusted hazard ratios (HRs) were estimated using Cox proportional hazards models controlling for age, sex, smoking status, family history of diabetes, and baseline body mass index (BMI). No statistically significant differences in diabetes incidence were observed across groups. Overall effect size for association between initial component and diabetes incidence: Cramer’s V = 0.018 (negligible effect). Abbreviations: O, abdominal obesity; BP, elevated blood pressure; T, high triglycerides; H, low HDL cholesterol; HR, hazard ratio; CI, confidence interval; MetS, metabolic syndrome.

## Data Availability

The data underpinning this study’s findings are accessible from the corresponding author upon reasonable request.
